# *DsFoxO* knockout affects development and fecundity of *Drosophila suzukii*

**DOI:** 10.3389/fphys.2023.1290732

**Published:** 2023-11-10

**Authors:** Shan Zhao, Ruijuan Wang, Yan Liu, Long Su, Xiaoyan Dai, Dongyun Qin, Hao Chen, Zhenjuan Yin, Li Zheng, Yifan Zhai

**Affiliations:** ^1^ Institute of Plant Protection, Shandong Academy of Agricultural Sciences, Jinan, China; ^2^ Key Laboratory of Natural Enemies Insects, Ministry of Agriculture and Rural Affairs, Jinan, China; ^3^ China Ministry of Agriculture and Rural Affairs (MARA) ‐ Centre for Agriculture and Bioscience International (CABI) Joint Laboratory for Bio‐Safety Shandong Sub‐Center, Jinan, China; ^4^ College of Agriculture, Guizhou University, Guiyang, China

**Keywords:** *D. suzukii*, *DsFoxO*, development, fecundity, transcriptome analysis

## Abstract

Forkhead box O (FoxO), a key transcription factor in many species, participates in numerous physiological and pathological processes of organisms through a variety of signaling pathways. In the present study, we established *DsFoxO* knockout (*DsFoxO*-KO) strain using CRISPR/Cas9, and the influence on development and fecundity of mutant strain were evaluated. To clarify the corresponding mechanism, a transcriptome analysis was conducted subsequently. The results showed that the survival rates of the *DsFoxO*-KO strain in larval, pupal, and adult stages were all significantly lower than those of control. The duration of the pupal stage was similar between the two strains; however, durations of egg, larva, adult preoviposition period (APOP), and total APOP (TPOP) in the *DsFoxO*-KO strain were all significantly longer compared to those of the control strain. The fecundity of the *DsFoxO*-KO strain was 20.31 eggs/female, which was significantly lower than that of the control strain (430.47 eggs/female). With the transcriptome analysis, 612 differentially expressed genes (DEGs) were identified. Following COG and GO analyses, we found that most of the DEGs were associated with the metabolic process. According to the KEGG database, the mTOR signaling, MAPK signaling, Wnt signaling, and Toll and Imd signaling pathways; insect hormone biosynthesis; autophagy; and apoptosis were altered in the *DsFoxO*-KO strain. These results demonstrated that knockout of *DsFoxO* in *D. suzukii* significantly influenced its development and fecundity, while transcriptome analysis provided insights to explore the corresponding molecular mechanism. These findings highlighted the critical role of FoxO in *D. suzukii* and might contribute to the development of novel management strategies for these flies in the future.

## Introduction

*Drosophila suzukii*, commonly known as spotted wing *Drosophila*, was first reported in Japan in 1931 as a cause of damage to fruit crops (Matsumura, 1931). Currently, the fly has spread across the world to more than 52 countries via commercial exchanges and tourism (Andreazza et al., 2017; Dos Santos et al., 2017; Little et al., 2017). *D. suzukii* is now considered a major agricultural pest species of thin-skinned fruits (Lee et al., 2011; Burrack et al., 2013; Mazzi et al., 2017). Unlike most *Drosophila* species that prefer to lay eggs on fermenting fruits, *D. suzukii* is a more serious threat as its special oviposition site selection (Atallah et al., 2014). *D. suzukii* females are able to pierce the skin of ripening healthy fruits with their enlarged ovipositor and deposit eggs inside the flesh (Poyet et al., 2015; Muto et al., 2018), causing evident damage before harvest. Additionally, other pathogens, such as bacteria, yeasts, and fungi, could infect the fruits via damaged skin, leading to further deterioration (Asplen et al., 2015). The primary and effective approach to control *D. suzukii* is the application of chemical insecticides; however, the emergence of insecticide resistance and the environmental unsustainability have hampered the control efficiency of chemical insecticides (Shaw et al., 2018; Shaw et al., 2019; Mastore et al., 2021). Thus, researchers are actively seeking new methods and searching for potential target genes that might provide insights to develop alternative control strategies (Alphey and Bonsall, 2018; Ni et al., 2021).

The forkhead box O (FoxO) protein, a highly conserved transcription factor, has been shown to participate in a variety of physiological and pathological processes, including longevity, growth, stress resistance, and metabolism (Greer et al., 2007; Puig and Mattila, 2011). Unlike mammals that have four kinds of *FoxO* genes (*FoxO1*, *FoxO3a*, *FoxO4*, and *FoxO6*), *Drosophila* has only one (*dFoxO*) (Kaestner et al., 2000; de la Torre-Ubieta et al., 2010). The DNA-binding domain of *dFoxO*, located in the N-terminal, has 45% identity with FoxO4, whereas 84% identity in the three α-helix regions. In the C-terminal, *dFoxO* has a higher Ser and Gln content than mammals (Puig et al., 2003). In *Drosophila melanogaster*, activated FoxO had been shown to extend the lifespan of the organisms (Alic et al., 2014) and regulate RNA interference, providing protection against RNA virus infection (Spellberg and Marr, 2015). In mammals, FoxO proteins are widely expressed in the brain, where they could mediate both neuroprotection and neurodegeneration (Yuan et al., 2009; Siegrist et al., 2010). Numerous studies have also been conducted on mice, and the results showed that constant expression of FoxO proteins would regulate the activity of follicular, suggesting that the proteins are also involved in fecundity (Pelosi et al., 2013; Barilovits et al., 2014). Similar functions of FoxO were also found in *Caenorhabditis elegans* (Michaelson et al., 2010; Hibshman et al., 2016). Given that FoxO plays such important roles in a variety of organisms, it emerges as a potential candidate for targeting genes in pest control strategies.

A detailed molecular mechanism of FoxO has been gradually uncovered through the dedicated efforts of researchers, and several signaling pathways have been proved to be involved in its function. Among these pathways, insulin/insulin-like growth factor (insulin/IGF) signaling seems to be studied the most for FoxO functions, and through that, FoxO has been found to regulate a number of target genes involved in metabolism and cell progression (Tatar et al., 2001; Yamamoto and Tatar, 2011; Martins et al., 2016). Nam et al. (2022) revealed that translational controlled tumor protein (Tctp) regulates cell growth by reducing cytoplasmic FoxO levels in *Drosophila*. In *Tribolium castaneum*, *Blattella germanica*, and *D. melanogaster*, the effects of FoxO on fecundity have been linked to the downregulation of the expression of vitellogenin (Sheng et al., 2011; Süren-Castillo et al., 2012; Yang et al., 2013). After a deep research on *Helicoverpa armigera*, Zhang et al. (2022) suggested that pupal diapause is induced by the decreases of transforming growth factor-beta (TGFβ), which was stimulated by the FoxO-activated ubiquitin-proteasome system (UPS). All the aforementioned results demonstrate that FoxO is involved in different signaling pathways in different species, and further studies are still required to uncover the specific pathways in which FoxO participates in *D. suzukii*.

In the present study, to explore the influence of FoxO on development and fecundity of *D. suzukii*, we knocked out the *DsFoxO* gene through a 17-bp deletion in this species using the method of CRISPR/Cas9. Subsequent evaluation revealed that the development and fecundity of flies were largely influenced in this knockout strain. To explore the corresponding molecular mechanism, transcriptome analysis was conducted to screen the related genes and signaling pathways. The findings of this study provide further evidence of FoxO’s influence on *D. suzukii* and offer potential insights for the development of sustainable strategies in the management of this pest species.

## Materials and methods

### Insect rearing

The *D. suzukii* colony was originally collected from cherry orchards in Tai’an (Shandong Province, China) in 2012 and identified as the TA2012 strain. The flies were maintained in our laboratory in a climate-controlled incubator at 25°C ± 1°C under the condition of the 16L:8D daylight cycle and 70%–80% relative humidity (Zhai et al., 2016).

### Preparation of sgRNA

**FIGURE 1:**
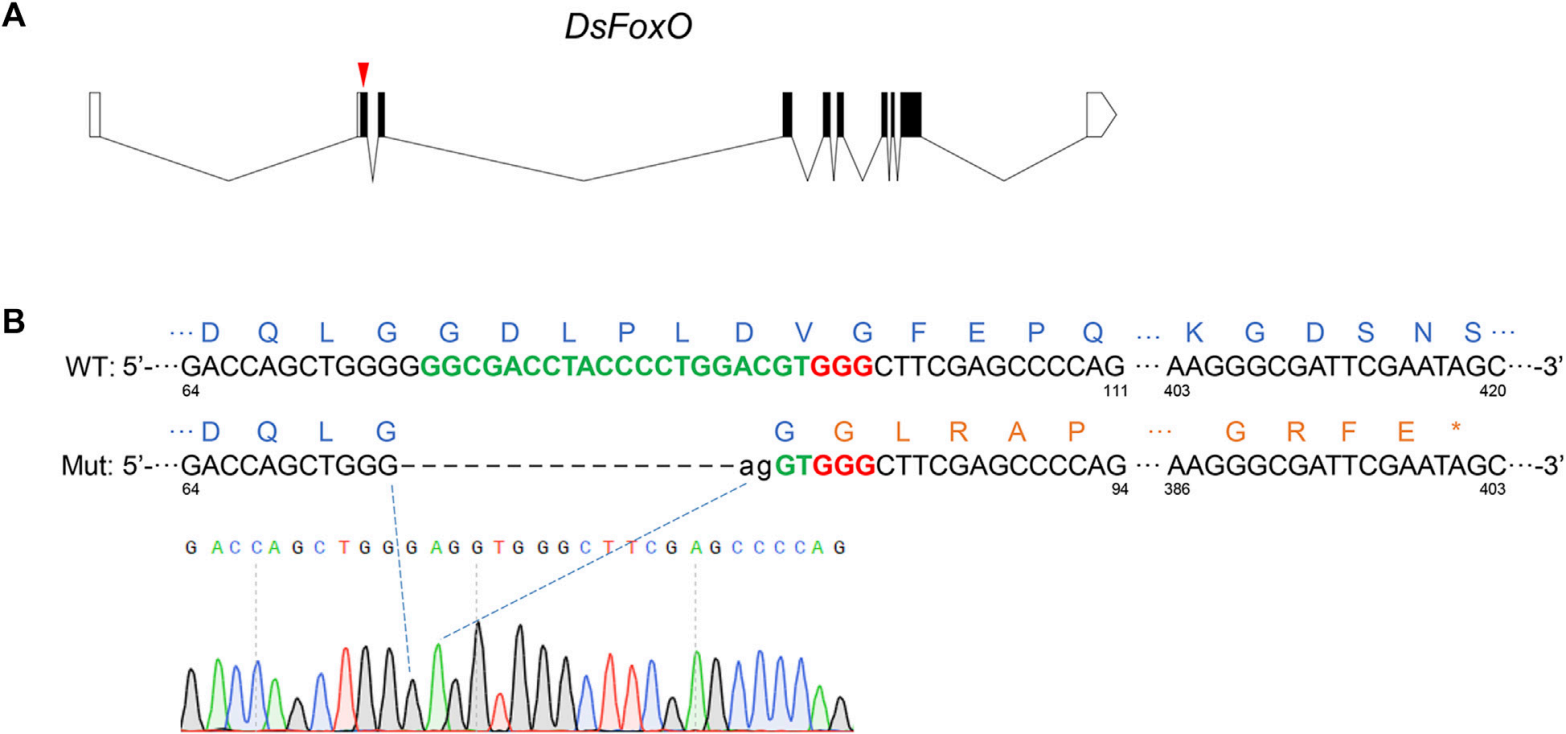
CRISPR/Cas9-mediated knockout of *DsFoxO* in *D. suzukii*. **(A)** Genomic structure of *DsFoxO* including protein-coding regions (black polygon) and untranslated regions (white polygon), and the position for sgRNA (red triangle). **(B)** Alignment analysis of PCR products from the wild-type (WT) strain and mutant (Mut) strain. Target sequences of sgRNA are in green with bold, while the PAM sequences in red with bold. Lowercase letters represent inserted bases. The amino acids of FoxO are in blue, while mutant amino acids are in brown. * indicates the terminator. The numbers represent the location of the base in the FoxO gene.

The single guide (sg)RNA target site of *DsFoxO* (XM_036818322.1) was identified with the principle of 5′-GGN_18_ **NGG**-3’ (the protospacer-adjacent motif (PAM) sequence is marked as bold). Gene structures are sketched in Figure 1A. The sgRNA used in this study was designed as 5′-GGC​GAC​CTA​CCC​CTG​GAC​GT**GGG**-3’. The sgRNA template was synthesized with two oligonucleotides, with one containing the T7 promoter and target sequence (5′-TAATACGACTCACTATAGGN_18_ GTTTTAGAGCTAGAAATAGC-3′) and the other was the universal oligonucleotide encoding the remaining sgRNA sequences (5′-AAAAGCACCGACTCGGTGCCACTTTTTCAAGTTGATAACGGACTAGCCTTATTTTAACTTGCTATTTCTAGCTCTAAAAC-3′). PCR, as well as the PCR product purification, was performed as described previously (Wang et al., 2016). Using purified DNA as templates, sgRNA was synthesized with the GeneArt™ Precision gRNA Synthesis Kit (Thermo Fisher Scientific, Vilnius, Lithuania), according to the manufacturer’s instruction.

### Embryo microinjection

Eggs, produced within half an hour, were collected and lined up on a microscope slide. Using the FemtoJet and InjectMan NI2 Microinjection system (Eppendorf, Hamburg, Germany), each egg was injected with 1 nl mixture of sgRNA (500 ng/μl) and Cas9 protein (250 ng/μl, Thermo Fisher, Shanghai, China). A total of 400 eggs were implemented with injection; after that, injected eggs were maintained in a climate-controlled incubator, as described previously.

### Identification of the *DsFoxO*-KO strain

Adults that hatched from injected eggs or resulted from the following generations were collected, and their genomic DNA was extracted using the AxyPrep™ DNA Extraction Kit (Axygen Biosciences, Union City, CA, United States) for genotyping. Using the extracted DNA as a template, PCR was performed with the primer pair (F: 5′-ATG​ATG​GAC​GGC​TTC​GCG​CAG​GAC​T-3′ and R: 5′-CTC​TGT​GGA​TGA​GAA​CCT​ACC​TC-3′). PCR products were sequenced by Tsingke Biological Company (Qingdao, China) to detect the indel mutation on the target site.

### Detection of life history traits

A total of 270 eggs of the *DsFoxO*-KO strain were collected and reared individually in Petri dishes (3.5 cm in diameter). The dishes were divided into three groups with each group containing 90 dishes. The duration of the egg incubation period, and larval and pupal stages were recorded daily, and the stage-specific survival rates (i.e., the proportion of individuals that survived from the previous stage) were calculated. Immediately after adult emergence, 15 male–female pairs were placed individually for mating in bottles. The bottles were divided into three groups with each group containing five bottles. The oviposition was observed over a period of 30 days, and the adult preoviposition period (APOP), total APOP (TPOP), and fecundity were recorded and calculated. The TA2012 strain was implemented with the same treatments and used as control.

### RNA sequencing

Total RNA of six adult females (15 days post-emergence) was extracted using TRIzol (Vazyme, Nanjing, China), following manufacturer’s instruction, and digested with DNase I (Vazyme, Nanjing, China). The mRNA with polyA tails was enriched with oligo (dT) magnetic beads and dealt with fragmentation buffer. cDNA was synthesized with random primers using mRNA as a template. After purification, the cDNA was repaired and A bases were added to 3′ ends. The cDNA sequences were screened with AMPure XP beads, and the cDNA library was obtained through PCR. After the library was confirmed as qualified, sequencing was performed with the Illumina NovaSeq6000 platform (BioMarker Technologies, Qingdao, China). Three biological replicates were used for RNA sequencing, and the TA2012 strain of *D. suzukii* was used as control.

### qRT-PCR

Six genes were selected randomly to verify the results of RNA sequencing. Total RNA of adults was extracted and treated with DNase I. cDNA was synthesized using the PrimeScript™ IV 1st strand cDNA Synthesis Mix (TaKaRa, Dalian, China) in a 20 μl reaction, containing 5×Mix 4 μl, Random 6-mers 2 μl, RNA 5 μg, and an appropriate amount of RNase-free dH_2_O, with a procedure of 30°C 10 min, 42°C 20 min, and 95°C 5 min. qRT-PCR was carried out using the 2×SYBR Green qPCR Mix (SparkJade, Qingdao, China) in a 20 μl reaction containing 2×SYBR qPCR Mix 10 μl, cDNA 2 μl, forward primer (10 μM) 0.4 μl, reverse primer (10 μM) 0.4 μl, ROX reference dye II 0.4 μl, and RNase-free H_2_O 6.8 μl, with a procedure of 95°C 20 s, 40 cycles of 95°C 3 s, and 60°C 30 s, and a dissociation stage of 95°C 15 s and 60°C 1 min. qRT-PCR was performed using the 7500 Fast Real-Time PCR System (ABI). The relative expression levels of genes were calculated using the 2^−ΔΔCt^ method, and *DsActin* was used as a comparator with the primer pair (F: 5′-CTA​CGA​GGG​TTA​TGC​CCT​GC-3′ and R: 5′-CGG​TGG​TGG​TGA​ACG​AGT​AA-3’).

### Statistical analysis

Data were analyzed by IBM SPSS Statistics 24, followed by separation of means ± SE using Student’s t-test. Differences were significant at *p* < 0.05.

## Results

### Establishment of the *DsFoxO*-KO strain of *D. suzukii*

To knockout *DsFoxO*, 400 eggs of the TA2012 strain were injected with sgRNA and Cas9, and 90 G_0_ embryos survived to adulthood. Of these, 72 were divided into eight groups for mating with each group containing six females and three males. After G_1_ eggs were collected, we genotyped the G_0_ adults for the expected CRISPR-mediated indel mutation with PCR and confirmed by direct sequencing of the PCR products. The results showed that 38 of these 72 adults were edited. Then, 20 G_1_ adults were collected and a single pair was carried out with the TA2012 strain. After G_2_ eggs were collected, the G_1_ adults were genotyped and the results showed that four adults were mutated. From the four mutants, we selected a heterozygous mutant with a 17-bp deletion (19 bp deleted and 2 bp inserted, Figure 1B) for further study. From the single-pair G_1_ family that had this one parent heterozygous, we obtained 65 adults (G_2_). G_2_ adults were genotyped with 1/4 wings, and the results showed that, among the 65 adults, seven females and six males were heterozygous. The 13 adults were pooled for mating to produce G_3_. G_3_ adults were also genotyped with 1/4 wings, and two females and one male were homozygous. These three adults were pooled to generate the *DsFoxO-*KO strain.

### Life history traits of the *DsFoxO*-KO strain

**FIGURE 2:**
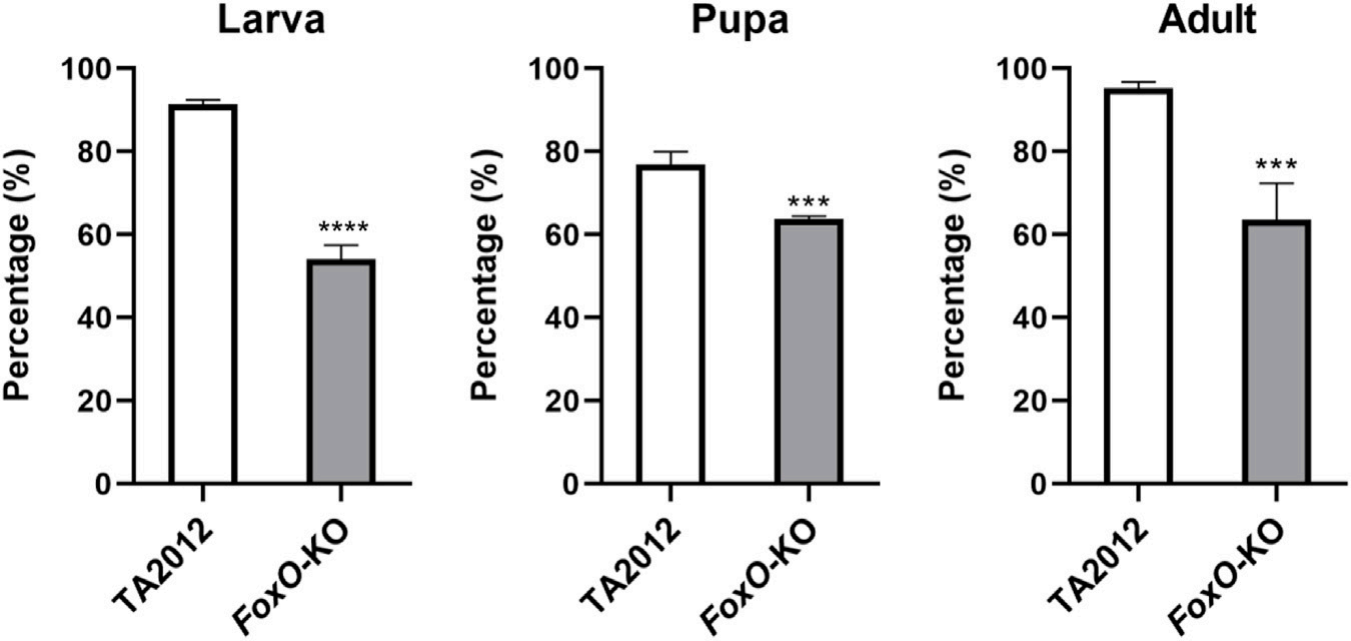
Survival rate of different life history stages of *DsFoxO*-KO and TA2012 strains in different stages. A total of 270 eggs were used and divided into three groups in this experiment. The data were shown as means ± SE. *** represents *p* < 0.005, and **** represents *p* < 0.001.

The effects of FoxO on life history traits in *D. suzukii* were detected in this study. The results showed that the survival rates of the *DsFoxO*-KO strain in the stages of larva, pupa, and adult were 54.07%, 63.69%, and 63.63%, respectively, which were all significantly lower than those in the TA2012 strain displayed as 91.33% (larva), 76.94% (pupa), and 95.27% (adult) (*p* < 0.05) (Figure 2).

**FIGURE 3:**
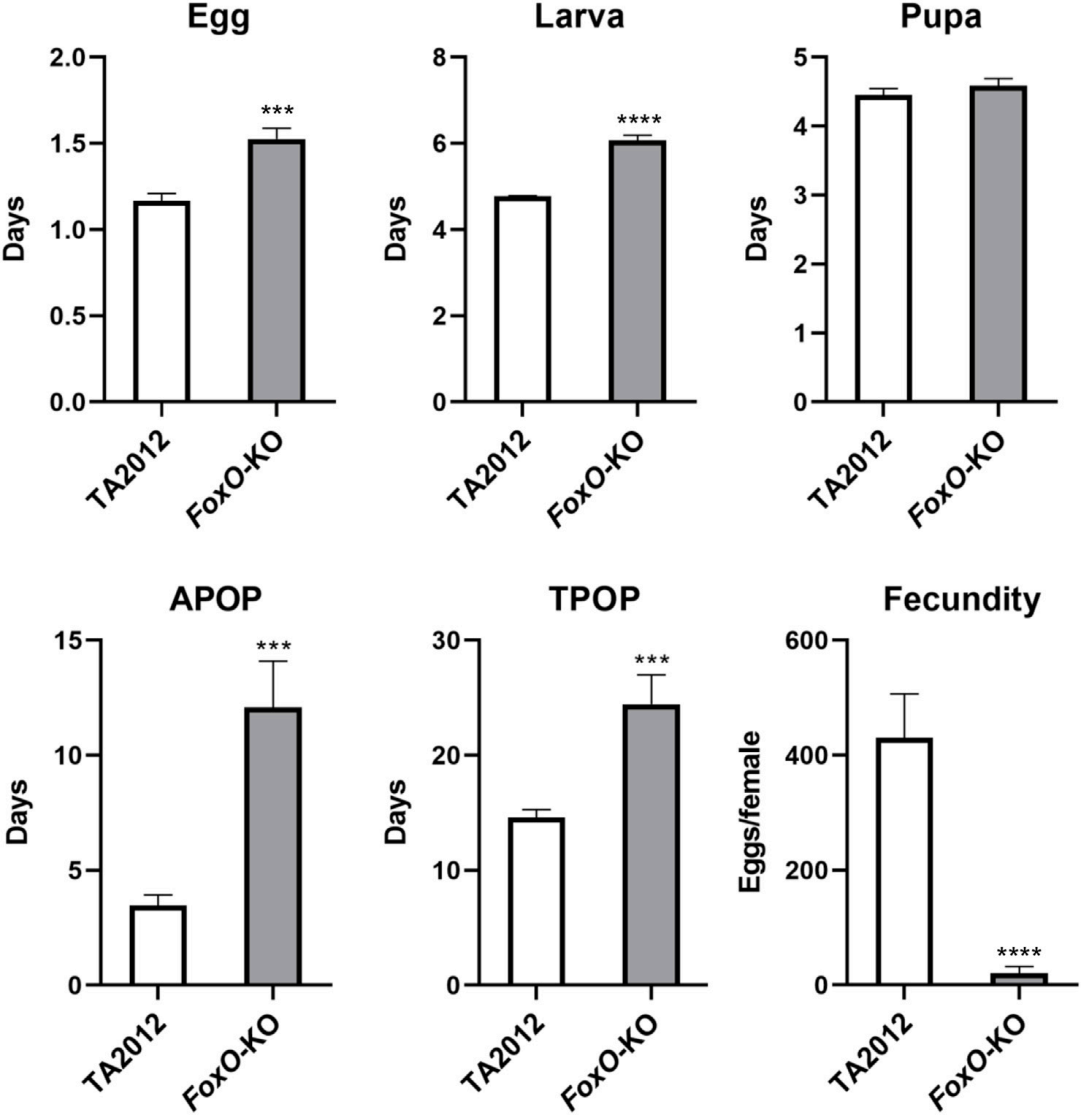
Comparison of life stage and fecundity parameters of *DsFoxO*-KO and TA2012 strains. The data were shown as means ± SE. *** represents *p* < 0.005, and **** represents *p* < 0.001.

The developmental periods of each stage were also detected in our study. The duration of the pupa stage was similar in the two strains (*p* > 0.05), whereas the egg and larval stages of the *DsFoxO*-KO strain (1.53 d and 6.07 d) were significantly longer than those in the TA2012 strain (1.17 d and 4.78 d) (*p* < 0.05). APOP and TPOP in the *DsFoxO*-KO strain were 12.08 d and 24.44 d, respectively, which were significantly longer than those in the TA2012 strain (3.47 d and 14.60 d) (*p* < 0.05). In 30 days after pairing, the fecundity in the *DsFoxO*-KO strain was 20.31 eggs/female, which was significantly lower than that in the TA2012 strain (430.47 eggs/female) (*p* < 0.05) (Figure 3).

### Differentially expressed genes (DEGs) resulting from *DsFoxO* knockout

Six samples (three replicates in each group) were used for RNA sequencing analysis, and low-quality and adapter sequences were removed from raw data using SOAPnuke software (v1.4.0) and Trimmomatic (v0.36). About 46.22 Gb clean data were obtained, with each sample containing clean data over 5.75 Gb. The clean reads in the *DsFoxO*-KO groups were 28882942, 26731905, and 29757666, respectively, and the percentages of GC contents were 54.30%, 54.39%, and 54.22%, respectively, while in the TA2012 strain, the clean reads were 20892031, 19266328, and 29112744 with the percentages of GC contents of 52.78%, 53.33%, and 54.62%, respectively. The percentages of bases with quality value ≥30 were all over 91.87% (Table 1).

**TABLE 1:** Statistics of RNA sequencing data.

Sample	Clean reads	Clean bases	GC content (%)	% ≥ Q30
*DsFoxO*-KO 1	28,882,942	8,634,060,946	54.30	93.27
*DsFoxO*-KO 2	26,731,905	7,994,123,456	54.39	92.63
*DsFoxO*-KO 3	29,757,666	8,897,339,064	54.22	92.73
TA2012 1	20,892,031	6,243,684,162	52.78	92.78
TA2012 2	19,266,328	5,754,641,294	53.33	94.03
TA2012 3	29,112,744	8,700,536,038	54.62	91.87

Clean reads, total number of paired-end reads in the clean data; GC content, percentage of G and C bases among the total bases; % ≥ Q30, percentage of bases with quality value ≥30.

**FIGURE 4:**
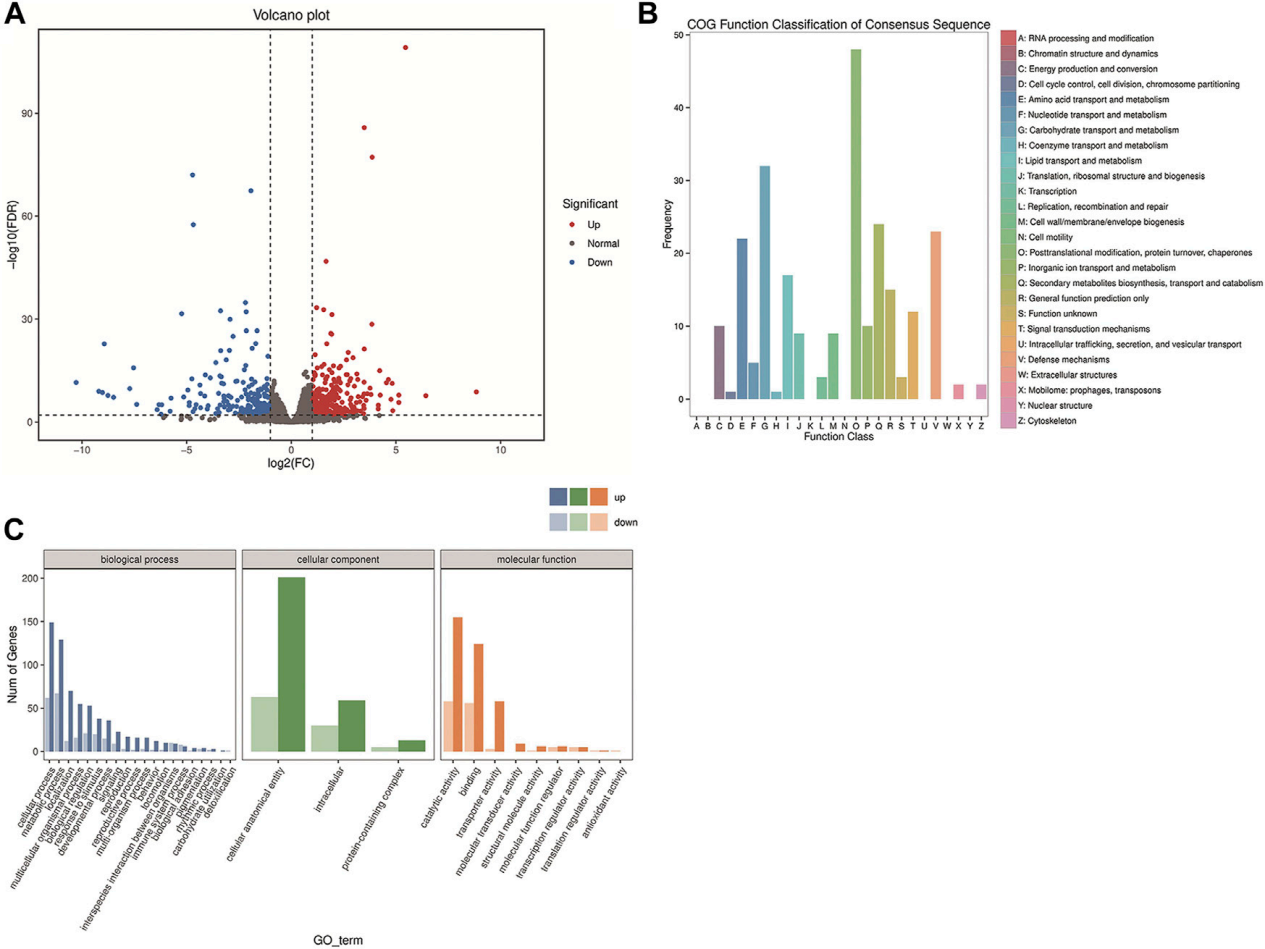
Transcriptome analysis of *DsFoxO*-KO and TA2012 strains. **(A)** Volcano plot of DEGs. Each dot represents a gene. The *X*-axis shows log2 (FC), and the *Y*-axis shows −log10 (FDR). All dots were filtered by FC ≥ 2 and FDR < 0.01. The red dots indicate upregulated DEGs. The blue dots indicate downregulated DEGs. The gray dots indicate normal genes. **(B)** Gene function classification based on the COG annotation. **(C)** Gene function classification according to GO assignments.

The expression levels of transcripts were quantified using the RSEM package (Bo and Dewey, 2011) and analyzed based on read counts with DESeq software (Love et al., 2014). The fold change (FC) ≥ 2 and false discovery rate (FDR) < 0.01 were used as standards, and FDR was classified by the *p*-value. In total, compared to the TA2012 group, 612 DEGs were identified, of which 404 were upregulated and 208 were downregulated (Figure 4A).

### DEG analysis

For functional annotation, all DEGs were analyzed with the methods of cluster of orthologous groups of proteins (COG) (Tatusov et al., 2000), the Gene Ontology (GO) consortium (Ye et al., 2006), and Kyoto Encyclopedia of Genes and Genomes (KEGG) database (Kanehisa et al., 2008).

The results of COG analysis showed that 248 sequences were classified to 26 categories with seven categories containing no sequences. Among these categories, the cluster for “Posttranslational modification, protein turnover, and chaperones” constituted the largest group (48, 19.35%), followed by “Carbohydrate transport and metabolism” with a number of 32 (12.90%) and “Secondary metabolites biosynthesis, transport, and catabolism” (24, 9.68%). On the contrary, “Cell cycle control, cell division, and chromosome partitioning” (1, 4.03%) and “Coenzyme transport and metabolism” (1, 4.03%) represented the smallest groups (Figure 4B).

The DEG enrichment was classified into 32 different groups, according to GO assignments, including three categories of biological process (20 groups), cellular component (three groups), and molecular function (nine groups). The biological process category accounted for the largest number of DEGs, among which the cellular process (211 DEGs) and metabolic process (196 DEGs) were the two most abundant terms. The cellular anatomical entity (264 DEGs) was the most abundant term in the cellular component category. In the molecular function category, catalytic activity (213 DEGs) and binding (180 DEGs) were the two most abundant terms (Figure 4C).

**FIGURE 5:**
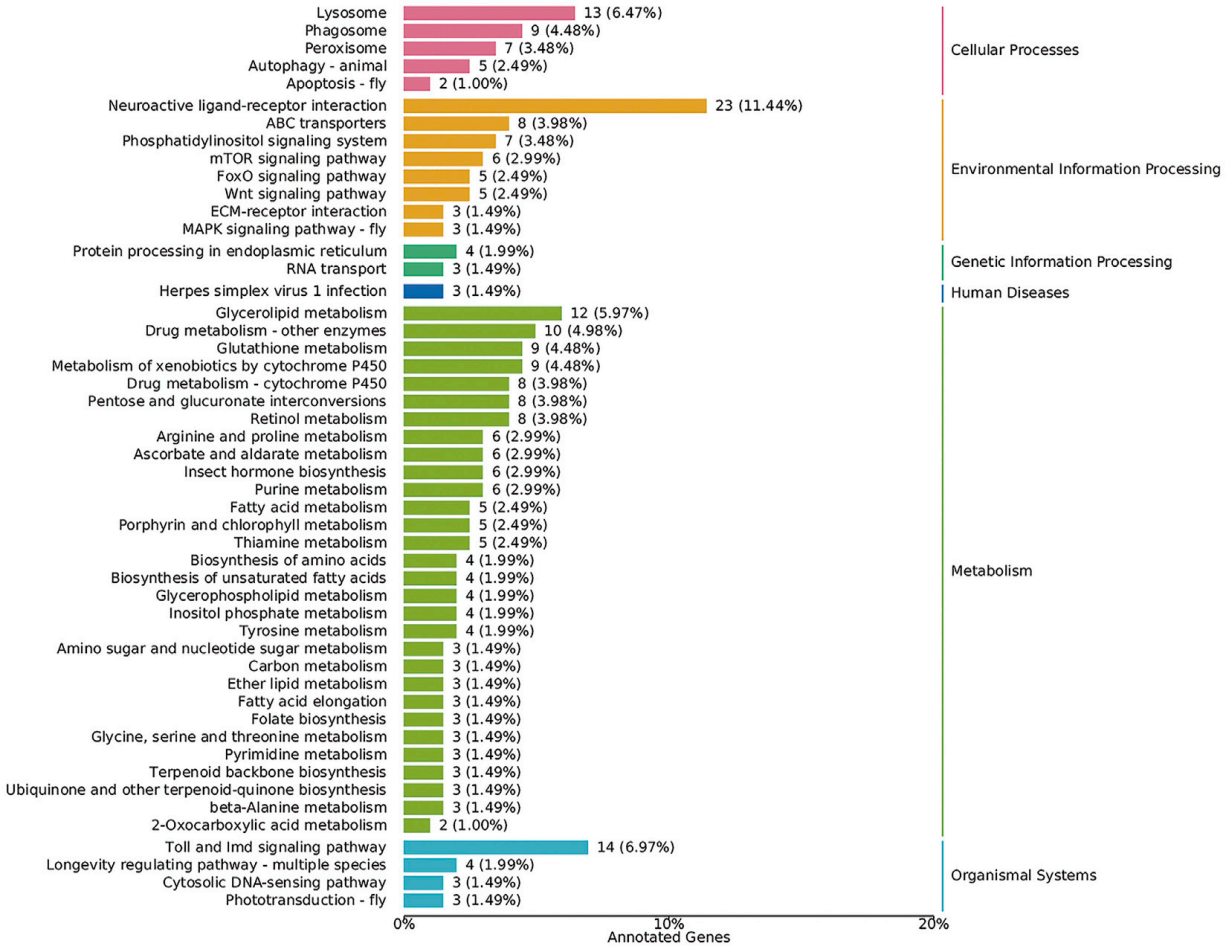
Biological pathways according to the KEGG database. The *X*-axis represents the percentage of genes. The *Y*-axis represents KEGG terms. Numbers on the right margin of each bar represent numbers of genes in the corresponding term.

The KOBAS database and clusterProfiler software were used to test the statistical enrichment of DEGs in KEGG pathways (Mao et al., 2005). In addition, 350 annotated genes were analyzed according to the KEGG database, and six biological pathways were identified, including cellular process, environmental information processing, genetic information processing, human diseases, metabolism, and organismal systems. Of these, the pathways represented by most genes were metabolism (155, 44.29%), followed by environmental information processing (60, 17.14%) (Figure 5).

**FIGURE 6:**
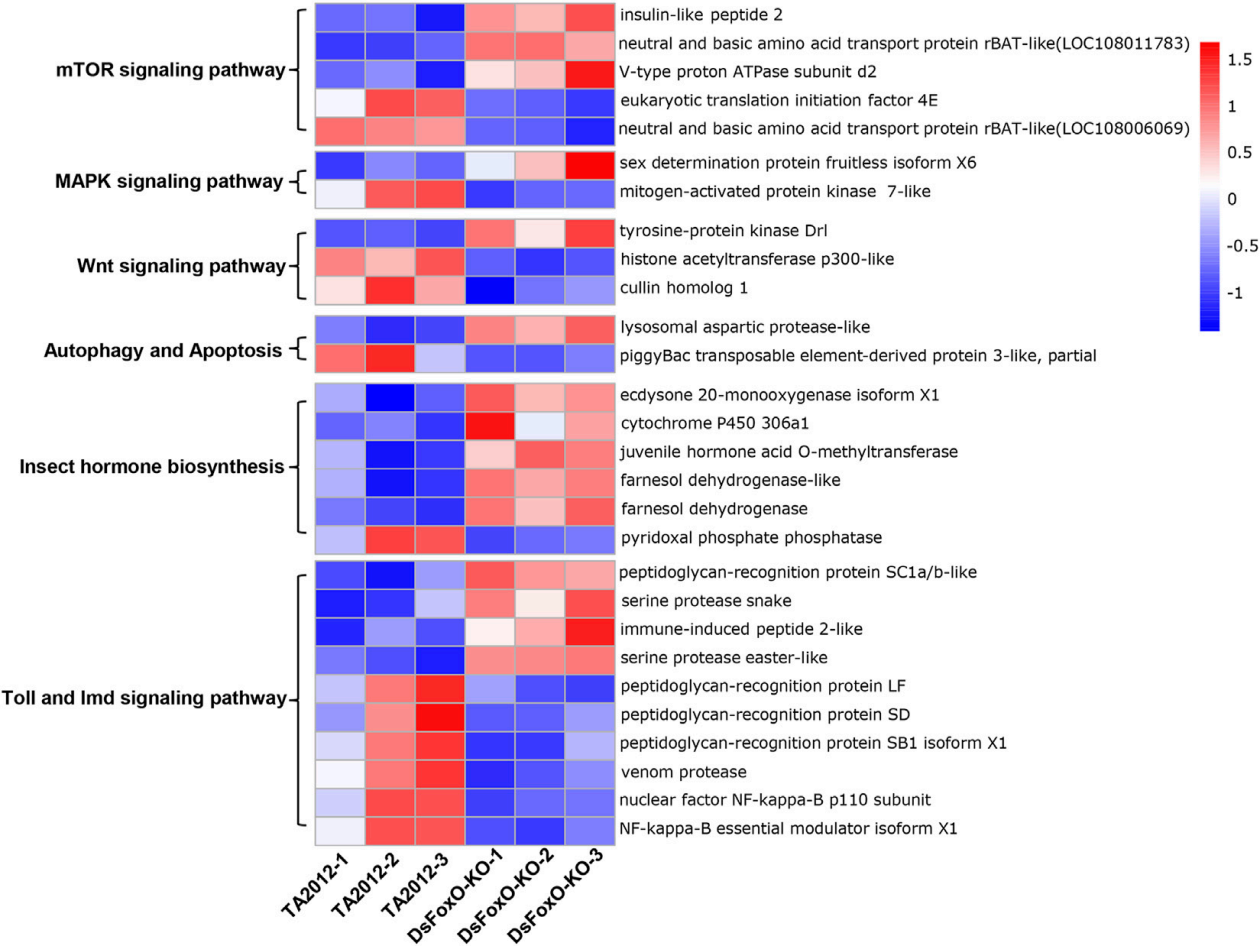
Expression heatmap of key genes in several pathways identified by KEGG. Red and blue indicate upregulated and downregulated genes relative to mean expression levels of the TA2012 strain, respectively, and the ratios were plotted on a color scale. Gene expression levels are represented by fragments per kilobase of transcript per million mapped reads (FPKM) and analyzed with log2 and Z-score.

In addition, the mTOR signaling pathway (six DEGs), MAPK signaling pathway (three DEGs), Wnt signaling pathway (five DEGs), Toll and Imd signaling pathways (14 DEGs), insect hormone biosynthesis (six DEGs), autophagy (five DEGs), and apoptosis (two DEGs) were all identified based on the KEGG database (Figure 5). The key genes in these pathways were analyzed, and we found that most genes involved in insect hormone biosynthesis were upregulated in the *DsFoxO*-KO strain, whereas genes in other pathways were mainly downregulated (Figure 6).

### Validation with qRT-PCR

**FIGURE 7:**
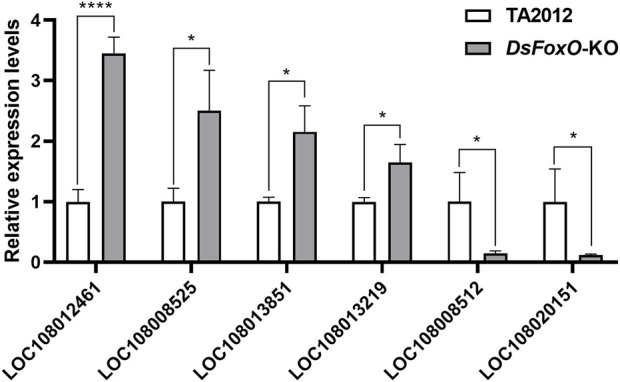
Expression level detection of six genes with qRT-PCR. The results were calculated using *DsActin* as control and were expressed as mean ± SE. * represents *p* < 0.05, and **** represents *p* < 0.001.

To validate the results of RNA sequencing, six genes were selected for qRT-PCR. The information about the genes and the primers is listed in Table 2. The results showed that the expression levels of LOC108012461, LOC108008525, LOC108013851, and LOC108013219 were upregulated, whereas those of LOC108008512 and LOC108020151 were downregulated compared with each gene in the TA2012 strain (Figure 7). The expression levels of all six genes were consistent with the results of RNA sequencing.

**TABLE 2:** Sequences of the primers used for the qRT-PCR analysis of six randomly selected genes.

Gene ID	Function	Primer (5′-3′)^a^	Log_2_(FC)
LOC108012461	Ecdysone 20-monooxygenase isoform (E20-MO)	AAC​ATA​AAA​GCG​CGC​CCC​AT	2.24
		CGG​AGG​TAA​ATC​TCC​GCC​AA	
LOC108008525	Juvenile hormone acid O-methyltransferase (JHAMT)	AGG​TGT​CAG​GAC​TGT​GAA​AGA	2.15
		GCC​CTG​CTG​CAA​ATT​CAT​TG	
LOC108013851	Farnesol dehydrogenase-like (FDH-like)	CCA​AGA​CCA​AGA​TTA​CGA​GCG	1.33
		CCA​TTG​GGT​TGC​TCC​CAA​GA	
LOC108013219	Insulin-like peptide 2 (ILP2)	GTC​CCT​CAT​CTC​GAT​GCT​CG	1.61
		GCC​TCT​CAC​CAC​AAC​GCT​GT	
LOC108008512	Hemolymph juvenile hormone-binding protein (JHBP)	TGT​TGC​AAC​GGC​TTT​GTC​TT	−3.19
		GGT​CTG​ATT​GTG​GAC​GGC​AA	
LOC108020151	Eukaryotic translation initiation factor 4E (eIF4E)	ACG​CTT​TGG​CTT​GTG​GAG​TA	−2.57
		TCC​AAA​AGT​CCT​CGA​CGG​TG	

^a^ Primers were tested and analyzed before the experiments.

FC, fold change.

## Discussion

As a key transcription factor, the functions of FoxO have been well studied in various species (Bai et al., 2013; Riedel et al., 2013). In *Drosophila*, FoxO has been demonstrated to play a role in metabolic adaptation, neuromuscular junction homeostasis, cytoskeletal dynamics, and microtubule functioning (Nechipurenko and Broihier, 2012; McLaughlin et al., 2016; Molaei et al., 2019; Birnbaum et al., 2021). In the present study, we aimed to explore the potential effects of FoxO on development and fecundity of *D. suzukii*. To obtain reliable experimental data and materials for further study, we knocked out the *DsFoxO* gene of *D. suzukii* using the method of CRISPR/Cas9. After selection for three generations, the mutant strain was genotyped as homozygous with 17 bp deletion, which changed the amino acid sequences from the indel site and terminated the mRNA at the location of 400 bp approximately (Figure 1B), indicating that the protein was knocked out completely. The establishment of the *DsFoxO*-KO strain laid the foundation for subsequent research.

FoxO has been previously suggested to affect the reproduction of female *Drosophila* (Yang et al., 2013). In our study, we assessed the reproduction of the *DsFoxO*-KO strain. In the *DsFoxO*-KO strain, fecundity was significantly reduced from 430.47 eggs/female in the TA2012 strain to 20.80 eggs/female, indicating that the reproduction of *D. suzukii* was influenced by FoxO. Similarly, FoxO was also reported to affect the number of eggs laid by female *Aedes aegypti* and *Nilaparvata lugens* (Hansen et al., 2007; Dong et al., 2021). In addition, the survival rates of *DsFoxO*-KO larvae, pupae, and adults were all significantly lower than those in the TA2012 strain. The duration of different life stages was also tested, and we found that the duration of egg, larval, APOP, and TPOP stages in the *DsFoxO*-KO strain were all significantly longer than those in TA2012 strain. These results suggested that FoxO would perform a huge impact on the development of *D. suzukii*, which were consistent with previous research studies (Alic et al., 2014; Dobson et al., 2019; Shi et al., 2019). Therefore, knocking out *DsFoxO* leads to a significant retardation in the development and fecundity of *D. suzukii*. These findings highlight the potential functions of FoxO in *D. suzukii*.

Cell homeostasis, which is determined by the metabolic steady state, is quite important for the development and fecundity of organisms. In *Drosophila*, as well as in mammals, FoxO can influence growth by regulating the cell cycle and energy metabolism (Jünger et al., 2003; Greer et al., 2007). Moreover, in differentiating cells, non-normal expression of FoxO would lead to cellular atrophy and promote a catabolic state (Accili and Arden, 2004). In the present study, after the transcriptome analysis was implemented, the data were analyzed with the methods of GO and KEGG. The results of GO analysis showed that DEGs were enriched in the cellular process, metabolic process, and catalytic activity, whereas most genes were presented in the metabolism pathway with KEGG analysis, suggesting that normal cellular and metabolic processes might be affected when FoxO was knocked out. These results were consistent with previous studies and suggested that knockout of FoxO in *Drosophila* might disrupt the cellular homeostasis, and thus, the development and fecundity of the flies were influenced. The development and fecundity of *Drosophila* would also be affected by the utilization of nutrition directly or indirectly as the energy for the organism was generated by various forms of nutrients (Ratnaparkhi and Sudhakaran, 2022). In COG analysis, numerous DEGs were classified into the category of “Carbohydrate transport and metabolism,” and these genes might restrict the availability of nutrients, which would also result in the alteration of development and fecundity of the flies.

Several pathways in which FoxO might be involved were also identified based on DEG analysis, including the mTOR signaling pathway, MAPK signaling pathway, Wnt signaling pathway, Toll and Imd signaling pathways, insect hormone biosynthesis, autophagy, and apoptosis (Figure 5). These aforementioned pathways all have been suggested to play important roles in insect growth and development (Zeng et al., 2017; Zhang and Zhang, 2019; Hughson et al., 2021). For example, alpha-ketoglutarate (AKG) would extend *Drosophila* lifespan by inhibiting the mTOR pathway (Su et al., 2019), whereas the development of *Drosophila* larvae was altered through the MAPK signaling pathway at the condition of ancestral dietary change (Towarnicki et al., 2022). Particularly, various DEGs were associated with the Toll and Imd signaling pathway, which is an important pathway in the innate immune response. DEGs in this pathway mainly encoded peptidoglycan recognition proteins (PGRPs) and nuclear factor kappa-B (NF-κB) proteins, which are the two key signaling molecules of the Toll and Imd pathways (Kawai and Akira, 2007; Royet and Dziarski, 2007), and both have also been proposed to play important roles in development processes (Maillet et al., 2008; Morgan and Liu, 2011). With a deep exploration of previous reports, we found that these pathways also regulated several physiological processes, including cell cycle, cell proliferation, apoptosis, and autophagy (Xing et al., 2015; Zeng et al., 2017; Maiese, 2021; Yan et al., 2022), all of which could influence the development and fecundity of the organism.

In terms of insect hormone biosynthesis classification, we observed that the expression levels of the ecdysone 20-monooxygenase isoform (E20-MO), juvenile hormone acid O-methyltransferase (JHAMT), and cytochrome P450 were all upregulated in the *DsFoxO*-KO strain. From the previous reports, we learned that the ecdysone biosynthesis mediated the *Drosophila* body size through a FoxO-Ultraspiracle interaction in case of nutritional control (Koyama et al., 2014). Similarly, Mirth et al. (2014) indicated that the effects of juvenile hormone (JH) on the growth rate in *Drosophila* are mainly dependent on FoxO. Although few studies have been reported on the relationship between FoxO and cytochrome P450, this protein had been reported to be essential for the synthesis and degradation of ecdysone and JH in insects (Bergé et al., 1998). Based on the results of our study, we hypothesized that, in *D. suzukii*, these hormones might impact the development and fecundity via their interactions with FoxO, and in the *DsFoxO*-KO strain, the growth of the flies were retarded to some extent, which warranted further investigation in future studies.

Based on the aforementioned information, we hypothesized that the functions of some hormones probably were limited without FoxO and that FoxO might influence the development and fecundity directly or via one or multiple pathways with the alteration of normal physiological processes. Perhaps, some of the pathways might modulate the development and fecundity by the reaction with FoxO. This hypothesis would be explored in future mechanistic studies. On the other hand, FoxO has also been implicated in diapause regulation in various species, and insulin signaling seems to be a key developmental pathway involved in this regulation (Tatar and Yin, 2001; Sim and Denlinger, 2013; Sim et al., 2015; Zhang et al., 2022). In our study, we also discovered that the expression level of insulin-like peptide 2 was upregulated in the *DsFoxO*-KO strain. Thus, further work is required on the relationship between diapause and FoxO in *Drosophila*.

In conclusion, we successfully established a *DsFoxO*-KO strain of *D. suzukii* using CRISPR/Cas9 in the present study. Life history trait analysis indicated that the development and fecundity were significantly influenced without FoxO. Through RNA sequencing, several DEGs were identified as their expression levels change or classification of pathways. Further studies with these DEGs might elucidate the molecular mechanism underlying the influence of FoxO on the development and fecundity of *D. suzukii*. Such insights will be useful for the development of strategies to control this economically important pest species.

## Data Availability

The datasets presented in this study can be found in online repositories. The names of the repository/repositories and accession number(s) can be found at: https://www.ncbi.nlm.nih.gov/, PRJNA1013261.
